# P02.55. Craniosacral therapy for migraine: a feasibility study

**DOI:** 10.1186/1472-6882-12-S1-P111

**Published:** 2012-06-12

**Authors:** J Mann, S Gaylord, K Faurot, C Suchindran, R Coeytaux, L Wilkinson, R Coble, P Curtis

**Affiliations:** 1 UNC School of Medicine, Chapel Hill, USA; 2 UNC Gillings School of Global Public Health, Biostatistics, Chapel Hill, USA; 3 Department of Community and Family Medicine, Duke University School of Medicine, Durham, USA; 4 Private practice, Carrboro, USA

## Purpose

The purpose of this study was to evaluate feasibility and obtain preliminary efficacy estimates comparing craniosacral therapy (CST) with an attention-control condition for the adjunctive treatment of migraine.

## Methods

Individuals with moderate to severe migraine were recruited from specialty clinics, family practices, and the university community. After confirmatory clinical evaluation and an 8-week run-in phase, those meeting study criteria (compliant with study procedures, at least 5 migraines per month) were randomized to 8 weekly CST or low-strength static magnet therapy (LSSM) treatments. Study participants were followed for 4 weeks after the conclusion of therapy. Primary outcome measures included headache frequency and headache-specific quality of life (HIT-6). Secondary headache-specific measures include headache-related disability (MIDAS), headache intensity, and abortive medication use.

## Results

At baseline, participants reported a mean 14 headache days per month and severe headache-related quality-of-life impact and disability. Compliance with study procedures was excellent, with 60 of 69 randomized individuals completing 8 weeks of therapy. Individuals in both treatment groups appeared to benefit from the therapy. A significant difference, favoring CST, was noted by treatment group in mean headache hours per day 30 days post treatment (1.89 vs. 2.78, p=0.003). HIT-6 scores decreased significantly in both groups, but without a between-group difference at the last treatment visit. MIDAS scores improved in the CST, but not the LSSM group at 4 weeks post treatment. Headache intensity was reduced more in the CST compared with the LSSM group, but the difference was not statistically significant. Abortive medication use decreased substantially in both groups during treatment.

## Conclusion

Our results show that conducting a randomized clinical trial of CST for migraine using a standardized protocol is feasible and that adjunctive CST may reduce headaches in those with severe migraine. Protocol modifications may enhance future investigations of CST for migraine.

